# A novel scoring system for precise severity quantification in severe fever with thrombocytopenia syndrome: development and application based on dynamic clinical data

**DOI:** 10.3389/fmicb.2026.1811615

**Published:** 2026-03-27

**Authors:** Yingchun Sun, Zhiyu Pan, Jing Sun, Yihui Sun, Wenjie Wang, Manman Liang, Aiping Zhang, Qiongle Wu, Haoyu Sheng, Jianghua Yang

**Affiliations:** 1 Department of Infectious Diseases, The First Affiliated Hospital of Wannan Medical College (Yijishan Hospital), Wuhu, Anhui, China; 2 Department of Cardiology, The First Affiliated Hospital with Nanjing Medical University (Jiangsu Province Hospital), Nanjing, Jiangsu, China

**Keywords:** application, dynamic, precise severity quantification, scoring system, SFTS

## Abstract

**Background:**

Severe Fever with Thrombocytopenia Syndrome (SFTS) is an acute infectious disease with high mortality. This study aimed to develop a quantitative scoring system for grading SFTS severity using dynamic clinical data.

**Methods:**

A retrospective study included 547 confirmed SFTS patients from two hospitals. Clinical data were collected over a 14-day course (divided into four phases). Patients were grouped into survivors (*n* = 451) and non-survivors (*n* = 96). Statistical analyses, including Kaplan–Meier curves and log-rank tests, were performed. A prospective external validation cohort of 44 newly diagnosed patients was subsequently enrolled to validate the scoring system using C-statistic, calibration curves, and decision curve analysis (DCA).

**Results:**

Of 547 patients, 96 (17.55%) were non-survivors. Multivariate logistic regression identified six independent prognostic factors across phases: age, platelet (PLT), aspartate aminotransferase (AST), and creatinine (Cr) (days 5–7); age, red blood cell distribution width (RDW), Cr, and lactate dehydrogenase (LDH) (days 8–10); Cr and LDH (days 11–14). A scoring system (0–11 points) was developed, stratifying patients into low (0–3), intermediate (4–7), and high (8–11) risk groups, with adverse outcome rates of 1.04, 22.92, and 76.04%, respectively. Kaplan–Meier curves showed significant prognostic differences (log-rank *p* < 0.001). External validation (44 cases) confirmed excellent performance: AUC 0.810–0.952, good calibration (Hosmer-Lemeshow *p* > 0.05), and net clinical benefit (DCA Eavg 0.068–0.098, Emax 0.422–0.559).

**Conclusion:**

A dynamic SFTS severity scoring system was developed and validated. Internal and external validation confirmed its reliability and clinical utility, providing a simple, practical tool for timely assessment and early intervention.

## 1 Introduction

Severe Fever with Thrombocytopenia Syndrome (SFTS) is an acute infectious disease caused by *Dabie bandavirus* (DBV), primarily transmitted via tick bites or contact with the blood and bodily fluids of infected individuals, and other routes (Liu et al., 2014; Peng et al., 2025; Sharma and Kamthania, 2021). Current research indicates that the case mortality rate of SFTS ranges from 12 to 30% in East Asia (Dualis et al., 2021; Li et al., 2018; Liu et al., 2014; Sun et al., 2025). The epidemic area continues to expand to new geographical regions, with both the proportion of severe cases and the mortality rate showing a gradual upward trend (Li et al., 2018; Liu et al., 2014; Sun et al., 2025). Thus, it has become a significant regional public health threat in China and several other East Asian countries (Dualis et al., 2021; Ning et al., 2023; JunWon et al., 2021; Sharma and Kamthania, 2021).

The clinical manifestations of SFTS are typically characterized by fever (often persistent high fever, with body temperature frequently ≥38.5 °C), thrombocytopenia, leukopenia, and gastrointestinal symptoms (such as nausea, vomiting, abdominal pain, and diarrhea). However, the severity of the disease varies considerably among patients. Some patients only present with mild, self-limiting symptoms, potentially accompanied by non-specific manifestations such as fatigue and muscle aches (Li et al., 2018; Li et al., 2022; Liu et al., 2014; Ning et al., 2023; Peng et al., 2025). These cases usually have a short course and favorable prognosis, resolving spontaneously without specific targeted treatment (Li et al., 2022; Ning et al., 2023). In contrast, other patients may rapidly progress to multi-organ failure (e.g., hepatic failure, renal failure, disseminated intravascular coagulation, shock), requiring urgent comprehensive resuscitative measures including mechanical ventilation, hemodialysis, and anti-shock therapy, yet still having a poor prognosis (Li et al., 2018; Li et al., 2022; Liu et al., 2014; Peng et al., 2025; Wang et al., 2021). Therefore, clinical practice requires individualized therapeutic approaches based on the severity of the patients’ conditions (Chen et al., 2025; Zhang et al., 2023; Zhong et al., 2024).

Currently, relevant domestic and international diagnostic and treatment guidelines have proposed preliminary frameworks for assessing the severity of SFTS, broadly classifying patients into mild and severe cases (Li et al., 2022; National Health and Family Planning Commission of the People’s Replublic China, 2010; Peng et al., 2025). However, the relevant criteria primarily rely on the presence or absence of clinical symptoms and thresholds for single laboratory indicators. For example PLT < 50 × 10^9^/L and white blood cell (WBC) counts<2 × 10^9^/L are used as reference indicators for determining severe cases (He et al., 2020; Li et al., 2016; Liu et al., 2014; Wang et al., 2022). Current research progress shows that there are significant shortcomings in the assessment SFTS severity, most notably the prevalence of cross-sectional study designs (Liu et al., 2024; Wang et al., 2024; Zhang et al., 2023; Zhong et al., 2024). Such studies typically collect laboratory indicators (e.g., PLT, WBC, hepatic and renal function, coagulation parameters) and clinical symptoms only at admission or a single specific time point. Constructing disease assessment models or screening for risk factors based on single-time-point data fails to reflect the dynamic progression of the disease (He et al., 2020; Wang et al., 2022). Without timely adjustments to monitoring frequency, this approach may delay intervention opportunities (Chen et al., 2025; Liu et al., 2024; Wang et al., 2024; Zhang et al., 2023; Zhong et al., 2024).

Therefore, to achieve a dynamic and precise quantitative assessment of disease severity in SFTS patients, this study divided the data of 547 SFTS patients within a 14-day disease course into four phases. By extracting the extreme values of indicators at each phase, the study captured the peak characteristics of disease severity, identified core risk factors for precise quantification, and established a stratified scoring system. External validation with new data provides clinicians with a dynamic, precisely quantifiable, and operationally feasible tools for SFTS disease grading.

## 2 Materials and methods

### 2.1 Data collection

A retrospective analysis was conducted on 547 confirmed SFTS patients admitted to the First Affiliated Hospital of Wannan Medical College (Yijishan Hospital) and the First Affiliated Hospital of Nanjing Medical University between May 2020 and June 2024. The patients were divided into a development set (464 cases) and an internal validation set (83 cases) in a ratio of 0.85:0.15 via simple random sampling (implemented in R software). A prospectively enrolled external validation set included 44 newly diagnosed SFTS patients from the First Affiliated Hospital of Nanjing Medical University between August 2024 and April 2025.

Inclusion criteria: ① Meeting the diagnostic criteria outlined in the Diagnosis and Treatment Protocol for Fever with Thrombocytopenia Syndrome (2020 Edition) (SFTSV nucleic acid positive or specific IgM antibody positive) (Liu et al., 2014; National Health and Family Planning Commission of the People’s Replublic China, 2010); ② Age≥18 years.

Exclusion criteria: ① Concurrent infectious diseases (e.g., influenza, sepsis); ② History of severe hepatic or renal insufficiency, hematological disorders, etc.; ③ Clinical data completeness rate<20%.

### 2.2 Staging of the disease

The course of SFTS was divided into 5 phases based on the dynamic change inflection points of laboratory indicators combined with clinical symptoms, in accordance with the research results of our team on the precise staging of SFTS (Hao et al., 2025; Hao et al., 2024):

Initial phase (days 1–4): The early stage of the disease with the onset of typical clinical manifestations of SFTS, without obvious organ dysfunction;

Progressive phase (days 5–7): The stage with the emergence of multi-organ damage manifestations and the initial occurrence of mortality, where the extent of organ dysfunction is aggravated and the number of prognostic risk factors increases significantly;

Multiple organ dysfunction (MOD) phase (days 8–10): The critical stage of SFTS with the highest mortality rate, featured by severe and obvious multi-organ dysfunction, which is the phase with the most pronounced differences in outcomes between fatal and non-fatal cases;

Remission phase (days 11–14): The stage with clinical improvement of patients, gradual relief of multi-organ dysfunction and a decrease in the number of prognostic risk factors, though the risk of adverse outcomes still exists;

Recovery phase (days 15–20): The stage with stable clinical conditions and gradual normalization of laboratory parameters in patients; this phase was not included in the subsequent statistical analysis of the present study.

### 2.3 Statistical methods

This study collected a total of 83 variables, including general information, epidemiological data, and laboratory indicators. With prognosis as the dependent variable and extreme values of indicators at various phases as independent variables, univariate logistic regression and LASSO regression were used during development phase to screen for potential prognosis-related variables. The selected variables were then incorporated into multivariate logistic regression analysis, with *p* < 0.05 serving as the inclusion criterion to determine independent risk factors.

Independent risk factors were incorporated into a nomogram, and ROC curves and the area under the ROC curve (AUC) were used to evaluate the predictive accuracy of the model. The overall efficacy of the model was validated through DCA.

In the development phase, the K-M curve was used to describe the cumulative incidence of adverse outcomes across risk strata, and the log-rank tests were used to compare differences between strata. The C-statistic was calculated to assess the discriminatory power of the scoring system.

In external validation, the C-statistic (95% confidence interval) was calculated to assess discriminatory ability; calibration curves was plotted and calibration was evaluated using the Hosmer-Lemeshow test (*p* > 0.05 indicates good calibration); DCA was constructed to compare the clinical net benefit of different scores.

Statistical analysis was performed using R 4.5.0(rms, glmnet, rmda, pROC, and insert packages) and SPSS Statistics 26.0. Quantitative data are presented as mean±SD or median, with intergroup comparisons performed using t-tests and Mann–Whitney U tests. Categorical data are expressed as frequencies, with intergroup comparisons conducted using χ^2^-tests. Two-tailed *p* < 0.05 was considered statistically significant.

## 3 Results

### 3.1 Baseline characteristics

We retrospectively enrolled 547 confirmed SFTS patients, among whom 244 (44.61%) were male and 303 (55.39%) were female, with a median age of 67 years (58–72 years). The overall mortality rate of this retrospective cohort was 17.55% (96/547). These 547 patients were further divided into a training set (*n* = 464) and an internal validation set (*n* = 83) via simple random sampling, and an additional 44 prospectively recruited SFTS patients were included as the external validation set, resulting in a total of 591 patients across the three cohorts. The detailed baseline characteristics of the three cohorts are summarized in Supplementary Table 1. No statistically significant difference was observed in age distribution among the training, internal validation and external validation sets (*p* = 0.376), suggesting excellent comparability in this key demographic characteristic. A statistically significant difference was detected in gender distribution across the three cohorts (*p* = 0.046), which was mainly attributable to the relatively lower proportion of female patients in the external validation set. Given the small sample size of the external validation set (*n* = 44), this gender imbalance is considered to stem from random variation rather than systematic bias. In addition, gender has not been identified as a major prognostic factor for SFTS in existing relevant literature and our internal analyses, so this minor imbalance does not compromise the overall comparability of the external validation set with the retrospective training and internal validation sets.

Dynamic analysis of clinical and laboratory indicators across four phases of the 14-day disease course revealed statistically significant differences in the distribution of core indicators between the survivor and non-survivor groups at each phase, with the number of differential indicators increasing first and then decreasing with disease progression (Table 1).

**Table 1:** Dynamic analysis of baseline characteristics between survivor and non-survivor groups by disease phases.

Date	Variables	Total (*n* = 547)	survival (*n* = 451)	non-survival (*n* = 96)	*p*-value
Day1-4	PLT	63(43, 84)	63(46.75, 84.25)	51(34, 77.25)	0.026
Day 5–7	Age	68(59, 72)	67(58, 71)	70(64.5, 77)	<0.001
	Lymph	0.6(0.4, 0.97)	0.6(0.42, 1)	0.44(0.34, 0.8)	0.004
	MONO	0.12(0.09, 0.3)	0.12(0.1, 0.3)	0.1(0.08, 0.23)	0.023
	RDW	13(12.6, 13.4)	13(12.5, 13.3)	13.1(12.7, 13.6)	0.041
	PLT	45.5(29, 62.5)	47.5(31, 65.25)	33(22.5, 47)	<0.001
	ALT	63.25 (43.35, 106.25)	63.25 (40.85, 99.65)	98.2 (63.25, 190.25)	<0.001
	AST	160.8 (94.5, 311)	153.3 (83.12, 257.22)	373.8 (160.8, 755.15)	<0.001
	BUN	6.42(5.08, 8.23)	6.42 (4.86, 7.78)	7.8(6.42, 11.96)	<0.001
	Cr	72.6(61.25, 88.65)	72.6(59.13, 83.82)	81.6(72.6, 121.25)	<0.001
	Ca	1.91(1.83, 2)	1.91(1.86, 2.01)	1.86(1.74, 1.94)	<0.001
Day 8–10	Age	67(58, 72)	66(57, 71)	70(63, 76)	<0.001
	WBC	6.09(3.77, 9.22)	5.78(3.7, 8.78)	7.55(4.8, 11.46)	0.005
	Neut	3.84(1.87, 6.78)	3.54(1.78, 6.47)	5.75(2.9, 8.57)	0.002
	MCV	90.6(87.7, 93.4)	90.6(87.62, 93.3)	91.3(88.8, 94.8)	0.032
	MCH	30.5(29.5, 31.5)	30.5(29.5, 31.4)	31.1(29.5, 32.1)	0.023
	RDW	13.2(12.7, 13.6)	13.1(12.6, 13.5)	13.6(13.2, 14)	<0.001
	PLT	44(28, 67)	46(30, 69)	29(23, 43)	<0.001
	MPV	12.2(11.2, 13.1)	12.2(11.3, 13.2)	11.8(10.9, 12.7)	0.012
	ALT	90(62, 133)	90(60.05, 122)	110(90, 204.8)	<0.001
	BUN	5.93(4.45, 8.61)	5.76(4.2, 7.38)	11.39(7, 16.93)	<0.001
	Cr	67(53.5, 82.6)	65.35(51.82, 75.3)	119.1(72, 162.7)	<0.001
	LDH	764.5 (498.5, 1,244)	764.5 (460, 1000.75)	1878 (897, 3,326)	<0.001
	CK	379 (195.5, 829.5)	379 (158.5, 635.25)	1,058 (379, 2,590)	<0.001
	ALB	30.8(28.7, 33)	30.8(29, 33.38)	30.7(27.5, 30.8)	0.002
	K	3.54(3.17, 3.90)	3.54(3.15, 3.84)	3.79(3.38, 4.16)	<0.001
	Ca	1.95(1.86, 2.04)	1.95(1.88, 2.04)	1.91(1.79, 2.00)	0.001
Day 11–14	WBC	6.62(4.57, 9.79)	6.5(4.54, 9.41)	9.71(5.1, 14)	0.013
	Neut	4(2.41, 7.05)	4(2.36, 6.6)	6.84(2.59, 10.83)	0.01
	MCH	30.5(29.3, 31.4)	30.5(29.3, 31.3)	30.85(29.42, 32.27)	0.045
	RDW	13.2(12.6, 13.7)	13.2(12.6, 13.7)	13.55(13.2, 14.43)	<0.001
	PLT	74(41, 112)	78(44, 116.5)	35(24.25, 59.75)	<0.001
	ALT	83(56, 127)	83(53, 123.9)	98.95(83, 192.5)	0.003

In the initial phase (days 1–4), only PLT showed an intergroup difference (*p* = 0.026), with the non-survivor group having a significantly lower median PLT level.

In the progressive phase (days 5–7), multiple indicators presented intergroup differences (all *p* < 0.05), among which age, PLT, AST, and Cr exhibited extremely significant differences (*p* < 0.001); the non-survivor group was characterized by older age, elevated AST and Cr, and reduced PLT.

The MOD phase (days 8–10) had the largest number of differential indicators, covering blood routine parameters, platelet parameters, hepatic and renal function indices, myocardial enzymes, and electrolytes. All indicators showed extremely significant differences (*p* < 0.001) except for mean corpuscular volume (MCV) and mean corpuscular hemoglobin (MCH) (*p* < 0.05), and the non-survivor group presented with elevated WBC, neutrophil (Neut), red blood cell distribution width (RDW), hepatic and renal function indices, myocardial enzymes, and decreased PLT.

In the remission phase (days 11–14), the number of differential indicators decreased, and only WBC, Neut, RDW, PLT, and alanine aminotransferase (ALT) still had intergroup differences (all *p* < 0.05), with the non-survivor group remaining with abnormal core indicators such as decreased PLT and elevated RDW.

### 3.2 Prognosis-related factors across disease phases

Univariate analysis revealed significant differences in factors associated with the prognosis of SFTS patients across different disease phases (Table 2).

**Table 2:** Screening of prognosis factors at different disease phases in SFTS patients.

Date	Variables	Coefficient	SE	OR	95%CI	*p*-value
Day 1–4	PLT	−0.015	0.007	0.985	(0.973, 0.998)	0.027
Day 5–7	Age	0.060	0.015	1.062	(1.030, 1.094)	<0.001
	PLT	−0.030	0.007	0.971	(0.958, 0.984)	<0.001
	ALT	0.003	0.001	1.003	(1.001, 1.005)	0.002
	AST	0.002	0.000	1.002	(1.001, 1.003)	<0.001
	BUN	0.161	0.031	1.175	(1.105, 1.249)	<0.001
	Cr	0.014	0.003	1.014	(1.008, 1.019)	<0.001
Day 8–10	Age	0.057	0.015	1.059	(1.029, 1.090)	<0.001
	WBC	0.055	0.022	1.057	(1.011, 1.104)	0.014
	Neut	0.053	0.026	1.054	(1.002, 1.109)	0.041
	MCH	0.146	0.072	1.157	(1.004, 1.333)	0.044
	RDW	0.487	0.125	1.627	(1.275, 2.078)	<0.001
	PLT	−0.026	0.006	0.974	(0.963, 0.985)	<0.001
	MPV	−0.205	0.081	0.815	(0.695, 0.956)	0.012
	ALT	0.002	0.001	1.002	(1.000, 1.003)	0.012
	AST	0.002	0.000	1.002	(1.001, 1.002)	<0.001
	BUN	0.088	0.020	1.092	(1.049, 1.136)	<0.001
	Cr	0.025	0.003	1.025	(1.018, 1.032)	<0.001
	LDH	0.001	0.000	1.001	(1.001, 1.001)	<0.001
	CK	0.000	0.000	1.000	(1.000, 1.001)	<0.001
	ALB	−0.106	0.034	0.899	(0.841, 0.961)	0.002
	K	0.367	0.177	1.443	(1.020, 2.041)	0.038
	Ca	−2.526	0.828	0.080	(0.016, 0.405)	0.002
Day 11–14	WBC	0.091	0.028	1.095	(1.037, 1.156)	0.001
	MONO	0.530	0.252	1.699	(1.037, 2.783)	0.035
	Neut	0.091	0.033	1.095	(1.028, 1.168)	0.005
	MCHC	0.028	0.011	1.028	(1.007, 1.050)	0.008
	PLT	−0.009	0.004	0.991	(0.984, 0.998)	0.015
	ALT	0.004	0.001	1.004	(1.001, 1.007)	0.006
	AST	0.004	0.001	1.004	(1.002, 1.005)	<0.001
	BUN	0.020	0.010	1.021	(1.001, 1.040)	0.035
	Cr	0.015	0.003	1.015	(1.010, 1.021)	<0.001
	LDH	0.001	0.000	1.001	(1.001, 1.001)	<0.001
	CK	0.000	0.000	1.000	(1.000, 1.000)	0.021

Days 1–4: Only PLT was associated with prognosis, indicating that decreased PLT levels during this phase are associated with an increased risk of prognosis.

Days 5–7: Six prognostic factors were identified, including age, PLT, ALT, AST, blood urea nitrogen (BUN), and Cr. Elevated levels of age, ALT, AST, BUN, and Cr, as well as decreased PLT, were all associated with prognosis.

Days 8–10: This phase exhibited the most abundant prognostic factors, totaling 16 items. These included blood count parameters [WBC, Neut, MCH, RDW, PLT, mean platelet volume (MPV)], hepatic and renal function indicators (ALT, AST, BUN, Cr), cardiac enzyme profiles, and metabolic markers [LDH, creatine kinase (CK), albumin (ALB), potassium (K), calcium (Ca)]. Among these, RDW, Cr, and LDH demonstrated strong predictive value for prognosis.

Days 11–14: Eleven indicators correlated with prognosis, including WBC, Neut, PLT, AST, Cr and LDH, among others, with particularly significant correlations observed for Cr and LDH.

### 3.3 Independent risk factors for prognosis

**Figure 1:**
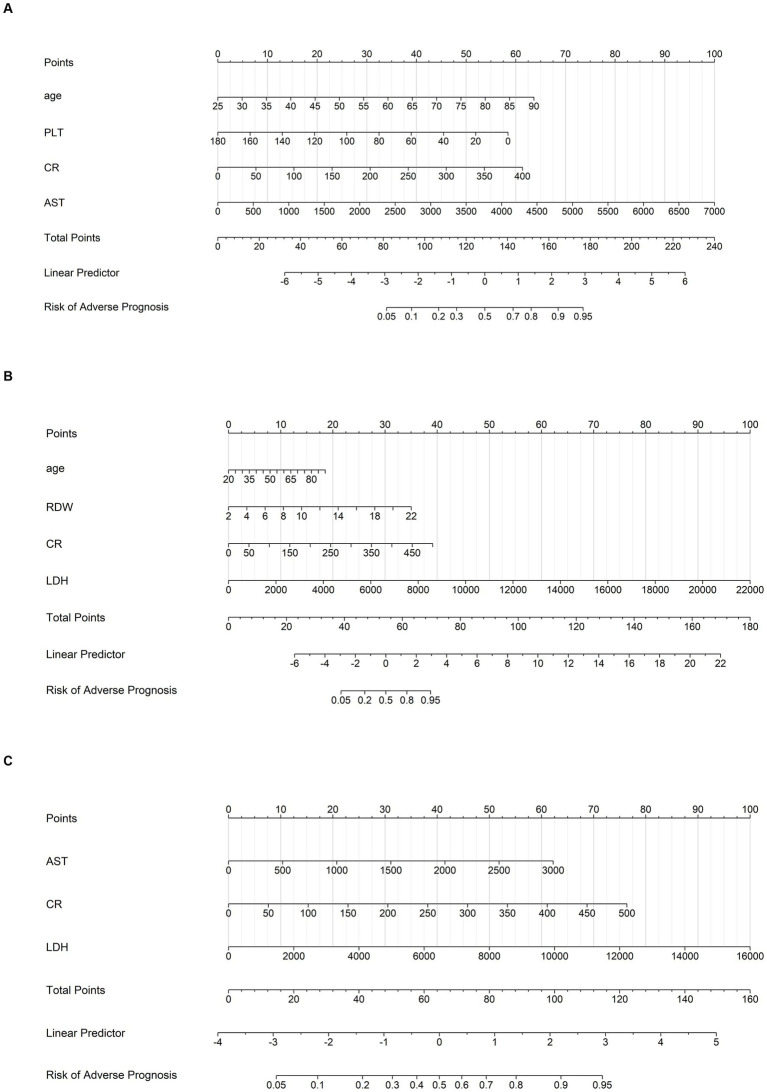
Nomograms. Nomograms for predicting the risk of adverse prognosis in patients with SFTS across different disease phases: **(A)** nomogram for predicting during the progressive phase; **(B)** nomogram for predicting during the MOD phase; **(C)** nomogram for predicting during the remission phase.

Multivariate logistic regression confirmed 6 independent risk factors: age, RDW, PLT, AST, Cr, and LDH. The composition of independent risk factors differed across disease phases (Table 3): Days 1–4: No clear independent risk factors were identified. Days 5–7: Age, PLT, AST and Cr were independent risk factors. Days 8–10: Independent risk factors included age, RDW, Cr, and LDH. Days 11–14: Only Cr and LDH were independent risk factors (Figure 1).

**Table 3:** Independent risk factors for prognosis at different phases in SFTS patients.

Date	Variables	Coefficient	SE	OR	95%CI	*p*-value
Day 1–4	–	–	–	–	–	–
Day 5–7	Age	0.0607	0.0191	1.0625	(1.0236, 1.1028)	0.0015
	PLT	−0.0201	0.0081	0.9801	(0.9644, 0.9961)	0.0127
	AST	0.0009	0.0004	1.0009	(1.0001, 1.0017)	0.0155
	Cr	0.0095	0.0030	1.0095	(1.0036, 1.0155)	0.0016
Day 8–10	Age	0.0504	0.0205	1.0517	(1.0105, 1.0940)	0.0140
	RDW	0.3496	0.1498	1.4185	(1.0573, 1.9038)	0.0196
	Cr	0.0134	0.0034	1.0135	(1.0068, 1.0202)	<0.0001
	LDH	0.0008	0.0002	1.0008	(1.0004, 1.0012)	<0.0001
Day 11–14	Cr	0.0090	0.0025	1.0090	(1.0041, 1.0140)	0.0003
	LDH	0.0004	0.0002	1.0004	(1.0000, 1.0008)	0.0496

### 3.4 Development of a severity scoring system for SFTS patients

Based on the clinical cut-off values and normal ranges, a stratified scoring rule was established (Table 4): the low-risk tier was assigned 0 points, the medium-risk tier was assigned 1 point, and the high-risk tier was assigned 2 points. For RDW, only low- and medium-risk tiers were assigned (≤14.5% scored 0 points, >14.5% scored 1 point), while all other indicators (PLT, AST, Cr, LDH, and age) were scored across three risk tiers. The total score ranges from 0 to 11 points, categorizing patients into low-risk (0–3 points), intermediate-risk (4–7 points), and high-risk (8–11 points) groups.

**Table 4:** Scoring rules and grading criteria for SFTS severity assessment.

Independent risk factors	0 points	1 points	2 points
RDW (%)	≤14.5	>14.5	–
PLT (×10^9^/L)	≥100	50–99	<50
AST (U/L)	≤40	40–292	>292
Cr (μmol/L)	≤81	81–103.7	>103.7
LDH (U/L)	≤250	250–1, 344	>1,344
Age (years)	≤67	67–73.5	>73.5
Overall score	0–3 points (low risk)	4–7 points (medium risk)	8–11 points (high risk)

### 3.5 Severity stratification and clinical outcomes

#### 3.5.1 Overall severity stratification at 14 days post-admission

Among 547 patients, 99(18.1%) were classified as low risk, 320(58.5%) as intermediate risk, and 128(23.4%) as high risk. The corresponding 14-day adverse outcome rates were 1.04%(1/99), 22.92%(22/320), and 76.04%(73/128), demonstrating a significant risk gradient difference (Table 5). Mortality was extremely low in the low-risk group (0–3 points), with only one death occurring in the 3-point subgroup. The intermediate-risk group (4–7 points) exhibited moderate mortality, with rates increasing slightly across subgroups as scores rose. The high-risk group (8–11 points) showed a markedly elevated mortality rate, with the 11-point subgroup reaching 100% mortality and all 8-10-point subgroups exceeding 50% mortality. This indicates a strong association between higher risk scores and prognosis.

**Table 5:** Risk Stratification scores of SFTS patients admitted for 14 days.

Stratification	Scores	Cases (*n* = 547)	Survivor (*n* = 451)	Non-survivor (*n* = 96)	Mortality rate
Low-risk	0	2	2	0	1 (1.04%)
	1	3	3	0	
	2	15	15	0	
	3	79	78	1	
Intermediate-risk	4	94	93	1	22 (22.92%)
	5	92	85	7	
	6	77	72	5	
	7	57	48	9	
High-risk	8	60	34	26	73 (76.04%)
	9	45	18	27	
	10	22	3	19	
	11	1	0	1	

#### 3.5.2 Dynamic severity stratification across disease phases

Risk stratification and mortality rates exhibit dynamic correlations across different disease phases (Table 6).

**Table 6:** Dynamic risk stratification scores of SFTS patients during different disease phases.

Date	Stratification	Scores	All (*n* = 547)	Survivor (*n* = 451)	Non-survivor (*n* = 96)	Mortality rate
Days 1–4	Low-risk	0	12	12	0	17(17.71%)
		1	24	22	2	
		2	26	21	5	
		3	28	18	10	
	Intermediate-risk	4	31	27	4	14(14.58%)
		5	22	16	6	
		6	12	9	3	
		7	7	6	1	
	High-risk	8	3	2	1	5(5.21%)
		9	5	1	4	
		10	1	1	0	
		11	0	0	0	
Days 5–7	Low-risk	0	4	4	0	8(8.33%)
		1	19	17	2	
		2	30	28	2	
		3	82	78	4	
	Intermediate-risk	4	74	67	7	40(41.67%)
		5	68	53	15	
		6	51	42	9	
		7	27	18	9	
	High-risk	8	27	12	15	26(27.08%)
		9	14	5	9	
		10	1	0	1	
		11	1	0	1	
Days 8–10	Low-risk	0	4	4	0	7(7.29%)
		1	19	19	0	
		2	41	37	4	
		3	89	86	3	
	Intermediate-risk	4	97	94	3	32(33.33%)
		5	72	63	9	
		6	76	67	9	
		7	34	23	11	
	High-risk	8	31	11	20	38(39.58%)
		9	14	4	10	
		10	8	0	8	
		11	0	0	0	
Days 11–14	Low-risk	0	1	1	0	4(4.17%)
		1	9	8	1	
		2	38	36	2	
		3	83	82	1	
	Intermediate-risk	4	104	101	3	18(18.75%)
		5	88	86	2	
		6	52	46	6	
		7	23	16	7	
	High-risk	8	16	5	11	20(20.83%)
		9	8	3	5	
		10	4	0	4	
		11	0	0	0	

Days 1–4: Low-risk stratum mortality rate 17.71%, intermediate-risk stratum 14.58%, high-risk stratum 5.21%. The association between risk stratification and mortality at this stage is relatively weak, potentially due to early-stage disease indicators not yet fully manifesting abnormalities.

Days 5–7: Mortality in the intermediate-risk group rose to 41.67%, while the high-risk group reached 27.08%. The low-risk group maintained a low mortality rate of 8.33%, indicating that changes in progressive indicators began to clearly reflect disease severity.

Days 8–10: High-risk group mortality peaked at 39.58%, intermediate-risk group at 33.33%, and low-risk group at only 7.29%. This phase saw concentrated manifestation of multi-organ dysfunction, with risk stratification demonstrating its most pronounced predictive value for prognosis.

Days 11–14: Mortality rates declined across all risk groups, with low-risk at 4.17%, intermediate-risk at 18.75%, and high-risk at 20.83%. This progression aligns with the pathological course of disease improvement and organ function recovery during the remission phase.

### 3.6 Survival analysis by severity stratification

**Figure 2:**
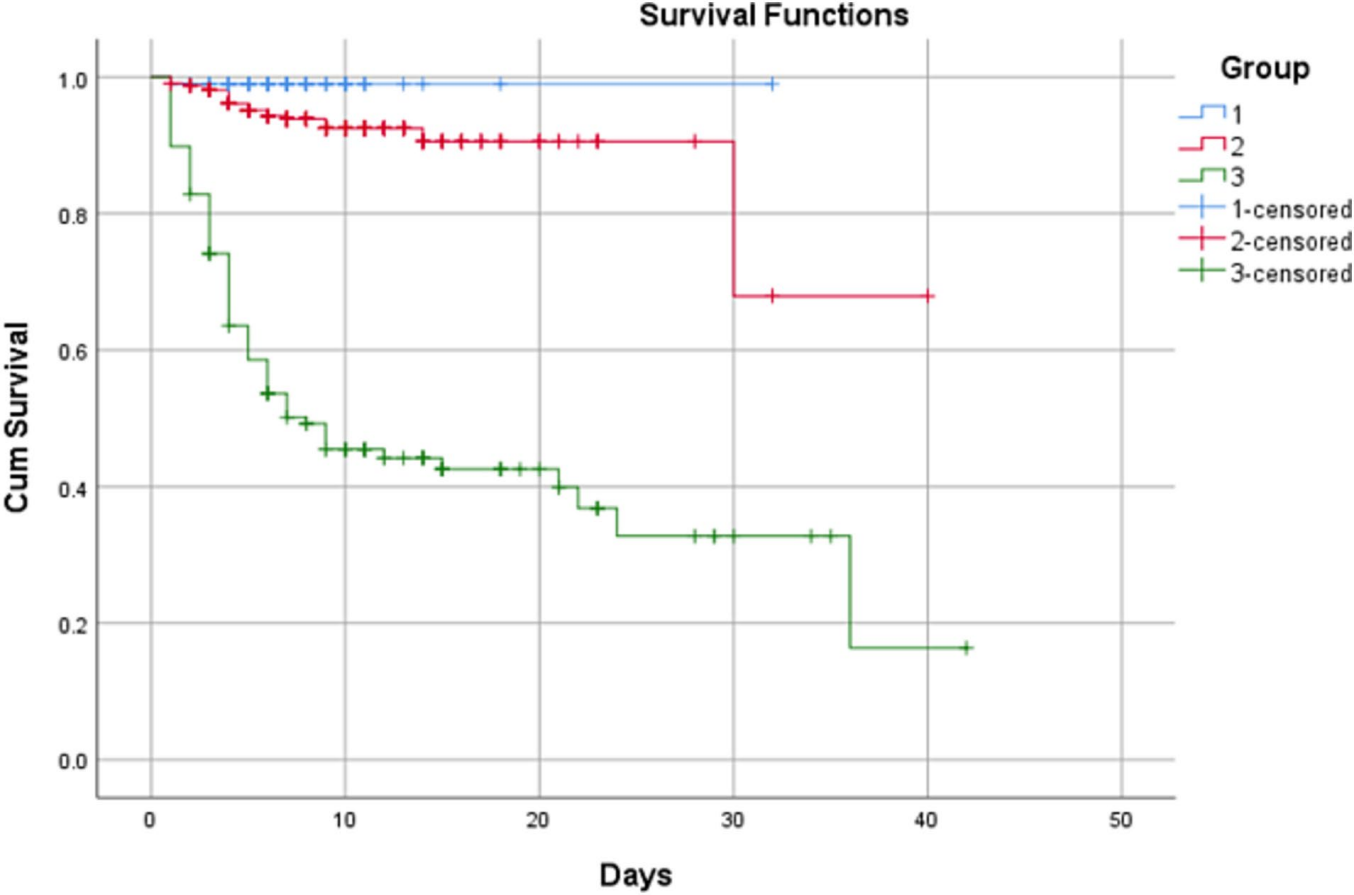
Kaplan–Meier curves for overall survival of SFTS patients stratified by the novel severity scoring system. (1) Low-risk; (2) intermediate-risk; (3) high-risk. Log-rank test result: χ^2^ = 163.211, degrees of freedom = 2, *p* < 0.001.

Survival analysis revealed (Table 7) that the overall survival rate in the low-risk group reached 99.00%, with a mean survival time of 31.684 days; the intermediate-risk group had an overall survival rate of 93.10% with a mean survival time of 34.598 days; while the high-risk group recorded an overall survival rate of merely 43.00%, with a mean survival time of 17.505 days and a median survival time of only 8 days. The log-rank test yielded χ^2^ = 163.658, degrees of freedom = 2, and *p* < 0.001, indicating statistically significant differences in survival curves among the low-, intermediate-, and high-risk groups. This demonstrates the scoring system’s effective risk stratification capability (Figure 2).

**Table 7:** Survival analysis of SFTS patients stratified by disease severity.

Stratification	Cases (n)	Non-survivor (n)	Survivor (n)	Overall survival rate (%)	Average survival time (day, 95%CI)	Median survival time (day, 95%CI)
Low-risk	99	1	98	99	31.687 (31.076–32.297)	–
Intermediate-risk	320	22	298	93.1	34.598 (30.520–38.676)	–
High-risk	128	73	55	43	17.505 (13.991–21.019)	8.000 (3.695–12.305)
All	547	96	450	82.4	29.994 (26.698–33.291)	36.000 (25.834–46.166)

Log-Rank test result: χ^2^ = 163.658, degrees of freedom = 2, *P* < 0.001 (indicating statistically significant differences among the three survival curves); “–” indicates that the median survival time was not reached for this group during the follow-up period (i.e., over 50% of patients survived beyond the upper limit of follow-up); 95% CI: 95% confidence interval.

### 3.7 Validation of the severity scoring system

Model validation results from the prospectively enrolled external validation cohort of 44 patients (Table 8) demonstrate the scoring system’s excellent predictive performance:

**Table 8:** Performance metrics of the SFTS severity scoring system across disease phases.

Date	AUC	Brier	Intercept	Slope	Emax	Eavg
Days 1–4	–	–	–	–	–	–
Days 5–7	0.810	0.138	0.917	1.591	0.448	0.098
Days 8–10	0.897	0.0504	0.0205	1.0517	0.422	0.097
Days 11–14	0.952	0.0004	0.0002	1.0004	0.559	0.068

**Figure 3:**
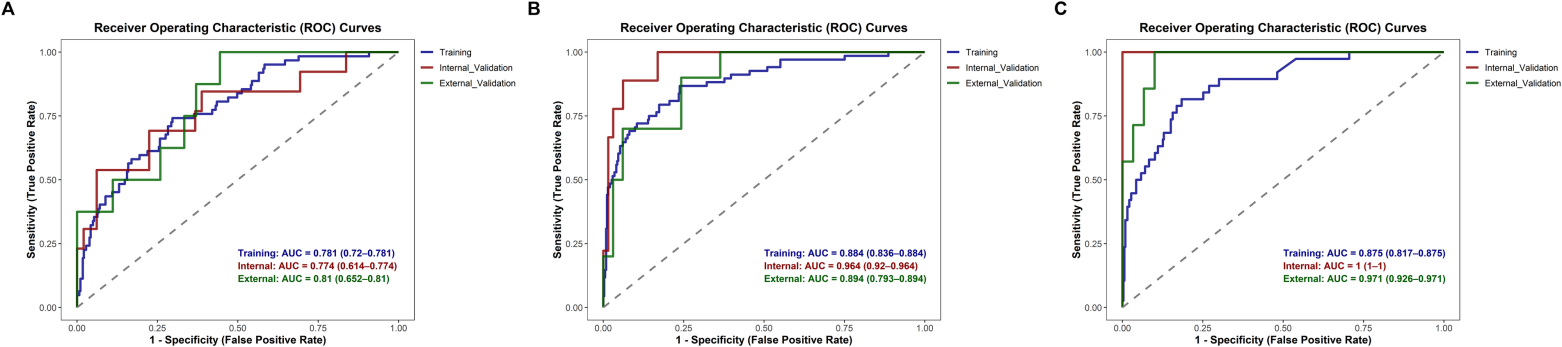
Receiver operating characteristic (ROC) curves. Receiver operating characteristic (ROC) curves for evaluating the discriminatory power of the SFTS severity scoring system across different disease phases: **(A)** progressive phase; **(B)** MOD phase; **(C)** remission phase.

Discrimination: AUC values ranged from 0.810 to 0.952 across disease phases, with predictive accuracy progressively improving as the disease advanced. Notably, the 11–14 day phase achieved an AUC of 0.952, indicating optimal model predictive efficacy during this phase (Figure 3).

**Figure 4:**
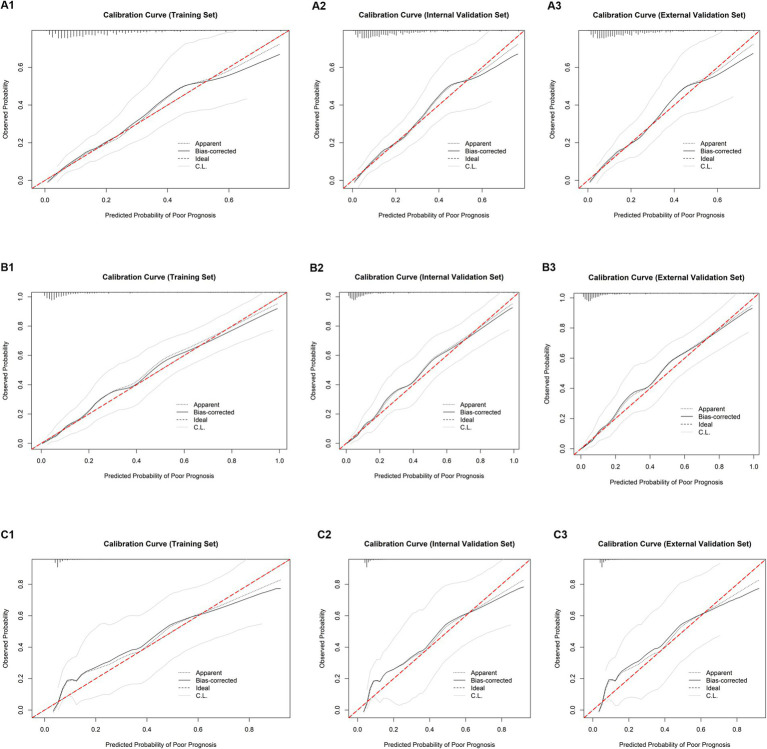
Calibration curves. Calibration curves for evaluating the concordance between predicted and actual poor prognosis probabilities of the SFTS severity scoring system across different disease phases: **(A)** progressive phase; **(B)** MOD phase; **(C)** remission phase; (1) Training set; (2) Internal validation set; (3) External validation set.

Calibration: The intercept and slope of the calibration curve fell within reasonable ranges, with the Hosmer-Lemeshow test *p* > 0.05, indicating high concordance between predicted probabilities and actual patients outcomes (Figure 4).

**Figure 5:**
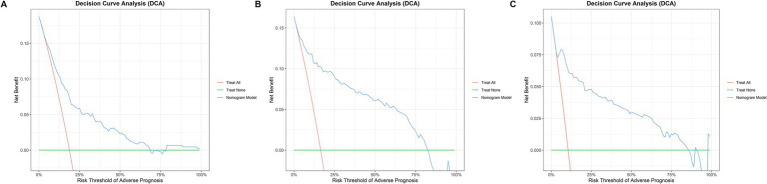
Decision curve analysis (DCA). Decision curve analysis (DCA) for evaluating the clinical net benefit of the SFTS severity scoring system across different disease phases: **(A)** progressive phase; **(B)** MOD phase; **(C)** remission phase.

Clinical net benefit: DCA revealed that DCA curves for all phases exceeded the extreme curve. The mean net benefit (Eavg) ranged from 0.068 to 0.098, with a maximum net benefit (Emax) of 0.422–0.559. This effectively mitigates clinical intervention bias, providing reliable support for clinical decision-making (Figure 5).

## 4 Discussion

Severe Fever with Thrombocytopenia Syndrome (SFTS) is an emerging acute infectious disease caused by DBV infection, primarily transmitted by tick bites but also through contact with bodily fluids and blood of SFTS patients (Liu et al., 2014; Peng et al., 2025; Sharma and Kamthania, 2021). It is characterized by high mortality rates and widespread transmission (Dualis et al., 2021; Li et al., 2018; Sun et al., 2025). The primary clinical symptoms of SFTS include fever, fatigue, digestive system symptoms, and hepatic and renal insufficiency. Severe cases may also present with central nervous system symptoms, disseminated intravascular coagulation (DIC), MOD, and even death (Liu et al., 2014; Ning et al., 2023; JunWon et al., 2021; Wang et al., 2021; Wang et al., 2022). Currently, no specific antiviral therapies are available for SFTS, and clinical management primarily relies on symptomatic and supportive care (Li et al., 2022; National Health and Family Planning Commission of the People’s Replublic China, 2010; Peng et al., 2025). Therefore, early identification of the disease severity and the implementation of stratified interventions are crucial for improving patients prognosis (Chen et al., 2025; Zhang et al., 2023; Zhong et al., 2024).

To date, several studies have developed prognostic assessment tools for SFTS, yet most rely solely on laboratory data at the single time point of hospital admission. These tools typically categorize results as normal or abnormal based solely on reference ranges, overlooking the dynamic progression of the disease (Liu et al., 2024; Zhang et al., 2023). Consequently, they fail to provide effective guidance for adjusting intervention strategies during the course of illness (Chen et al., 2025; He et al., 2020; Li et al., 2016; Wang et al., 2022; Zhong et al., 2024). For instance, some patients admitted in the early disease phase may exhibit no typical abnormalities in relevant indicators, leading to misclassification as low-risk. Conversely, others experiencing rapid indicator changes during the mid-disease phase may suffer treatment delays due to the absence of dynamic monitoring (Wang et al., 2021; Wang et al., 2024; Zhang et al., 2023).

This study divided the 14-day disease course into four key phases, extracting the extreme values of laboratory findings at each phase (such as the lowest platelet count and peak AST levels) to more precisely capture the patterns of disease progression (Chen et al., 2025; He et al., 2020; Li et al., 2022; Liu et al., 2024; Wang et al., 2022). The pathophysiological process of SFTS is dynamically evolving: the initial phase is characterized by systemic inflammatory responses triggered by viral replication; the intermediate phase involves progressive platelet destruction and organ dysfunction (such as hepatic and renal insufficiency); the late phase may result in fatal outcomes due to severe inflammatory storms or multiple organ failure (Jin et al., 2012; Li et al., 2018; Liu et al., 2014; Peng et al., 2025; Sun et al., 2025; Yang et al., 2018; Yu, 2018). Findings measured at a single time point (e.g., upon admission) reflect only the disease state at that specific phase. This approach may yield inaccurate assessments if patients exhibit no typical abnormalities during the early disease phase or if indicators temporarily decline during compensatory periods (Liu et al., 2024; Wang et al., 2024; Zhang et al., 2023; Zhong et al., 2024).

Through LASSO regression and multivariate logistic regression analysis, we dynamically identified six independent prognostic risk factors—age, RDW, PLT, AST, Cr, and LDH—with distinct distributions across disease phases (He et al., 2020; Li et al., 2016; Liu et al., 2024; Wang et al., 2022). Elderly patients (age>67 years) exhibit compromised immune function and diminished viral clearance capacity, rendering them more susceptible to severe disease progression (Chen et al., 2017; Dualis et al., 2021; Hao et al., 2024; Li et al., 2018; Wang et al., 2021). Reduced PLT counts indicate significant excessive platelet consumption; prior studies suggest this may relate to transient suppression of bone marrow hematopoiesis induced by viral infection. Additionally, the binding of PLT to SFTSV further promotes their clearance by splenic macrophages, thereby increasing the patients’ risk of hemorrhage and MODS (Chen et al., 2017; He et al., 2020; Jin et al., 2012; Li et al., 2016; Wang et al., 2022). Elevated AST and LDH reflect hepatocyte and cardiomyocyte injury, serving as biomarkers of worsening hepatic damage and systemic inflammatory response (Hao et al., 2024; Shao et al., 2015; Wang et al., 2017; Wang et al., 2022; Zhang et al., 2023). Increased Cr levels are indicative of renal impairment, a critical hallmark of multi-organ failure in severe SFTS (Jin et al., 2012; Yang et al., 2018). Elevated RDW suggests abnormal erythropoiesis, which correlates with systemic inflammation and poor prognosis, potentially reflecting the indirect effects of viral-induced inflammation on bone marrow function (Dualis et al., 2021; Liu et al., 2024; Yang et al., 2018; Yu, 2018).

A key innovation of this study lies in its refined scoring methodology. Unlike conventional scoring systems that adopt arbitrary equal-point allocation without accounting for the clinical relevance of indicator ranges, we utilized the Youden index from ROC curves to determine evidence-based clinical cut-off values for each risk factor (He et al., 2020; Liu et al., 2024; Zhang et al., 2023; Zhong et al., 2024). Integrating these cut-offs with clinically validated normal reference ranges, we developed a stratified scoring rule that links point weights to the severity of clinical risk. For example, a PLT count <50 × 10^9^/L (high risk) was assigned 2 points, 50–100 × 10^9^/L (medium risk) 1 point, and 100–300 × 10^9^/L (low risk) 0 points. This approach ensures that the scoring system is both scientifically rigorous and clinically meaningful, avoiding over- or under-weighting of indicators based on arbitrary criteria.

To enhance clinical applicability, we further stratified total scores (0–11 points) into low-risk (0–3 points), intermediate-risk (4–7 points), and high-risk (8–11 points) tiers based on quartile analysis. Primary healthcare institutions can rapidly complete clinical assessments in three steps: assigning scores to indicators by tier, summing total scores, and performing risk stratification. This enables early evaluation of SFTS patients’ conditions. Unlike previous complex prognostic models (such as multivariate nomograms) (Chen et al., 2025; Wang et al., 2024; Zhang et al., 2023; Zhong et al., 2024), this scoring system is more concise, straightforward and practical, addressing the unmet need for simple tools in primary care settings (Peng et al., 2025; Sun et al., 2025). It provides a useful tool for stratifying SFTS severity and facilitating timely intervention.

The reliability and clinical utility of the scoring system were validated through a comprehensive multidimensional evaluation. In the development cohort, K-M curves and log-rank tests demonstrated statistically significant differences in mortality across risk tiers (log-rank *p* < 0.001), confirming the system’s ability to distinguish between patients with varying disease severity. In the external validation cohort, the C-statistic, calibration curves, and DCA collectively validated the systems robust discriminatory power (AUC = 0.810–0.952), excellent calibration (Hosmer-Lemeshow test *p* > 0.05), and substantial clinical net benefit (Eavg = 0.068–0.098, Emax = 0.422–0.559). Importantly, the DCA curves consistently outperformed extreme strategies (treating all or no patients as high-risk), effectively mitigating the risks of over-intervention (unnecessary intensive care for low-risk patients) and under-intervention (delayed treatment for high-risk patients) in clinical decision-making (Chen et al., 2025; Peng et al., 2025; Wang et al., 2024; Zhong et al., 2024).

Compared with existing SFTS scoring tools, our dynamic scoring system offers several key advantages. By incorporating phase-specific extreme values and evidence-based cut-offs, it more accurately reflects the dynamic progression of SFTS, enabling timely adjustments to intervention strategies (Liu et al., 2024; Zhang et al., 2023; Zhong et al., 2024). This has significant implications for clinical practice: Low-risk patients may undergo outpatient follow-up and home isolation, minimizing unnecessary healthcare resource utilization and avoiding overtreatment (Chen et al., 2025; Peng et al., 2025). Intermediate-risk patients require close monitoring of dynamic changes in indicators (such as daily re-evaluation of PLT, hepatic function and renal function), with timely adjustments to treatment regimens and symptomatic management (He et al., 2020; Li et al., 2016; Wang et al., 2017; Wang et al., 2022). High-risk patients necessitate early initiation of intensive care, including immunomodulatory therapy, supportive treatment (such as platelet transfusion), and hepatoprotective and nephroprotective interventions, to reduce the risk of MODS and improve survival (Chen et al., 2017; Dualis et al., 2021; JunWon et al., 2021; Wang et al., 2021).

Notably, our findings highlight that the MOD phase (days 8–10) is the most critical period for prognosis, with the highest number of independent risk factors and peak mortality rates (He et al., 2020; Liu et al., 2024; Wang et al., 2021). This underscores the importance of intensified monitoring and intervention during this phase, as timely organ support may prevent irreversible multi-organ damage (Chen et al., 2017; Dualis et al., 2021). Additionally, the identification of Cr and LDH as persistent independent risk factors throughout the progressive, MOD, and remission phases emphasizes the central role of renal and myocardial injury in SFTS prognosis, providing targets for focused organ protection (Hao et al., 2024; Li et al., 2016; Li et al., 2018; Peng et al., 2025; Wang et al., 2022).

This study also has certain limitations. The retrospective design of the primary cohort analysis may introduce inherent selection and information bias, inter-assay variability in the detection of laboratory indicators could also exert a potential influence on the study results, and the impact of variations in patient treatment adherence on clinical outcomes was not evaluated in the present study. Missing data were imputed using median imputation in this research, and while this approach effectively reduced data loss, it may have introduced a certain degree of statistical error; the accuracy of subsequent research findings could be further improved by optimizing the standardization of clinical data collection and record-keeping practices. In addition, several potential confounding factors such as antiviral drug administration were not included in the current analysis, and expanding the range of incorporated variables including specific treatment regimens, troponin levels and viral load in future studies would help to further optimize the constructed scoring system and enhance its comprehensive prognostic value. Furthermore, the prospectively enrolled external validation cohort consisted of a relatively small sample size, and the generalizability and external validity of this SFTS severity scoring system could be significantly strengthened by conducting multicenter studies with larger and more diverse sample sizes for further external validation.

## 5 Conclusion

Age, RDW, AST, LDH, Cr, and PLT constitute independent risk factors for prognosis in SFTS patients. This study developed and validated a dynamic, phase-specific scoring system for SFTS severity quantification, based on 14-day clinical data and evidence-based cut-offs. The system effectively stratifies patients into low-, intermediate-, and high-risk tiers, with robust reliability and substantial clinical utility. By enabling early, precise risk assessment and individualized intervention, this tool addresses an unmet clinical need in SFTS management, potentially improving patients prognosis and optimizing healthcare resource allocation.

## Data Availability

The data analyzed in this study is subject to the following licenses/restrictions: The data that support the findings of this study are available from the corresponding author upon reasonable request. Requests to access these datasets should be directed to JY, yjhpath@163.com.
