# Supplementary material for: A novel scoring system for precise severity quantification in severe fever with thrombocytopenia syndrome: development and application based on dynamic clinical data
Source: Front Microbiol. 2026 Mar 27;17:1811615. doi: 10.3389/fmicb.2026.1811615 (PMC13066260; doi:10.3389/fmicb.2026.1811615)
Supplement: Supplementary file 1 [file Table_1.docx]

****Appendix:Laboratory Parameters and Reference Ranges Used in This Study****

| Characteristic | Normal Range |
| --- | --- |
| ALB (Albumin) | 40-55 g/L |
| ALT (Alanine Aminotransferase) | 7-40 U/L |
| APTT (Activated Partial Thromboplastin Time) | 23.3-32.5 s |
| AST (Aspartate Aminotransferase) | 15-35 U/L |
| BUN (Blood Urea Nitrogen) | 2.3-7.1 mmol/L |
| Ca (calcium) | 2.11-2.52=-0.41mmol/L |
| CK (Creatine Kinase) | 26-140 U/L |
| CKMB (Creatine Kinase MB) | 0-25 U/L |
| Cr (Creatinine) | 40-130 μmol/L |
| K (potassium) | 3.5-5.5 mmol/L |
| Na (Sodium) | 135-147 mmol/L |
| Cl (Chloride) | 99-110 mmol/L |
| D-D(D-Dimer) | 0-0.5 mg/L |
| LDH (Lactate Dehydrogenase) | 135-225 U/L |
| Lymph (Lymphocyte Count) | 0.8-4.0 ×10^9^/L |
| MCH (Mean Corpuscular Hemoglobin) | 27-34 pg |
| MCV (Mean Corpuscular Volume) | 80-100 fL |
| MONO (Monocytes) | 0.12-0.8 ×10^9^/L |
| MPV(Mean Platelet Volume) | 7-11 fL |
| Neutro(Neutrophils) | 2.5-7 ×10^9^/L |
| PLT(Platelets) | 100-300×10^9^/L |
| RDW (Red Cell Distribution Width) | 11.5-14.5 % |
| WBC(White Blood Cells) | 4-10 ×10^9^/L |
| PCT (Procalcitonin) | 0-0.5 ng/mL |
| CRP (C-Reactive Protein) | 0-5 mg/L |
| PT (Prothrombin Time) | 9-14 s |
| INR (International Normalized Ratio) | 0.8-1.2 |
| TBIL (Total Bilirubin) | 4-26 μmol/L |
| DBIL (Direct Bilirubin) | 1.17-14 μmol/L |
| cTnI (Cardiac Troponin I) | 0-0.03 ng/mL |
| UA (Uric Acid) | 90-420 μmol/L |
| PCT (Procalcitonin) | 0-0.5 ng/mL |
