# Supplementary material for: A novel scoring system for precise severity quantification in severe fever with thrombocytopenia syndrome: development and application based on dynamic clinical data
Source: Front Microbiol. 2026 Mar 27;17:1811615. doi: 10.3389/fmicb.2026.1811615 (PMC13066260; doi:10.3389/fmicb.2026.1811615)
Supplement: Supplementary file 2 [file Table_2.docx]

### **Supplemental Table 1. Baseline characteristics of patients with SFTS in the training, internal validation and external validation cohorts**

| **Characteristic** | **Total**  **(N=591)** | **Training**  **(N=464)** | **Internal_Validation**  **(N=83)** | **External_Validation**  **(N=44)** | **P-Value** |
| --- | --- | --- | --- | --- | --- |
| **Age, years** | 67(58,72) | 67(60,74) | 69(61,77) | 68.5(60.5,76.5) | 0.376 |
| **Sex, n(%)** |  |  |  |  | 0.046 |
| **female** | 319(54.0) | 255(55.0) | 48(57.8) | 16(36.4) |  |
| **male** | 272(46.0) | 209(45.0) | 35(42.2) | 28(63.6) |  |
